# Genetic diversity of *Plasmodium falciparum* in human malaria cases in Mali

**DOI:** 10.1186/s12936-016-1397-0

**Published:** 2016-07-11

**Authors:** Cécile Nabet, Safiatou Doumbo, Fakhri Jeddi, Salimata Konaté, Tommaso Manciulli, Bakary Fofana, Coralie L’Ollivier, Aminata Camara, Sandra Moore, Stéphane Ranque, Mahamadou A. Théra, Ogobara K. Doumbo, Renaud Piarroux

**Affiliations:** UMR MD3 IP-TPT, Parasitology Laboratory, Timone Hospital, Aix-Marseilles University, Marseilles, France; Malaria Research and Training Centre, Parasitic Diseases Epidemiology Department, UMI 3189, University of Sciences, Technique and Technology, Bamako, Mali; Department of Clinical, Surgical, Diagnostic and Paediatric Sciences, Division of Infectious and Tropical Diseases and Hepatology, University of Pavia, Pavia, Italy

**Keywords:** Malaria, *Plasmodium falciparum*, Mali, Genetic diversity, Epidemiology, MLVA

## Abstract

**Background:**

In Mali, *Plasmodium falciparum* malaria is highly endemic and remains stable despite the implementation of various malaria control measures. Understanding *P. falciparum* population structure variations across the country could provide new insights to guide malaria control programmes. In this study, *P. falciparum* genetic diversity and population structure in regions of varying patterns of malaria transmission in Mali were analysed.

**Methods:**

A total of 648 blood isolates adsorbed onto filter papers during population surveillance surveys (December 2012–March 2013, October 2013) in four distinct sites of Mali were screened for the presence of *P. falciparum* via quantitative PCR (qPCR). Multiple loci variable number of tandem repeats analysis (MLVA) using eight microsatellite markers was then performed on positive qPCR samples. Complete genotypes were then analysed for genetic diversity, genetic differentiation and linkage disequilibrium.

**Results:**

Of 156 qPCR-positive samples, complete genotyping of 112 samples was achieved. The parasite populations displayed high genetic diversity (mean He = 0.77), which was consistent with a high level of malaria transmission in Mali. Genetic differentiation was low (F_ST_ < 0.02), even between sites located approximately 900 km apart, thereby illustrating marked gene flux amongst parasite populations. The lack of linkage disequilibrium further revealed an absence of local clonal expansion, which was corroborated by the genotype relationship results. In contrast to the stable genetic diversity level observed throughout the country, mean multiplicity of infection increased from north to south (from 1.4 to 2.06) and paralleled malaria transmission levels observed locally.

**Conclusions:**

In Mali, the high level of genetic diversity and the pronounced gene flux amongst *P. falciparum* populations may represent an obstacle to control malaria. Indeed, results suggest that parasite populations are polymorphic enough to adapt to their host and to counteract interventions, such as anti-malarial vaccination. Additionally, the panmictic parasite population structure imply that resistance traits may disseminate freely from one area to another, making control measures performed at a local level ineffective.

**Electronic supplementary material:**

The online version of this article (doi:10.1186/s12936-016-1397-0) contains supplementary material, which is available to authorized users.

## Background

*Plasmodium falciparum*, which is transmitted by mosquitoes of the genus *Anopheles*, is the most common and the deadliest human malaria parasite. Despite extensive malaria elimination efforts, 214 million cases of malaria and 438,000 deaths were estimated globally in 2015, which primarily affected the sub-Saharan African population and children under 5 years of age [1]. Control strategies include the distribution of long-lasting insecticide-treated bed nets (LLINs), improved diagnosis using malaria rapid diagnostic tests (RDTs) and wider availability of artemisinin-based combination therapy (ACT). Even if control strategies have been intensified during the past 10 years in Mali, *P. falciparum* malaria remains highly endemic; stable incidence of the disease has been reported in several malaria vaccine-testing sites, such as Bandiagara [2]. In contrast to the situation in Mali, recent studies have shown that in other countries of the Sahel region, such as Senegal [3–5], enhanced interventions have effectively reduced malaria transmission. The heterogeneous results of malaria control programmes highlight the complexity of malaria epidemiology and the necessity to adapt interventions to local epidemiological settings.

In Mali, from the Sahara Desert to the Sudano-Guinean savannah, throughout the Sahel region, the variety of malaria transmission pattern is characterized by a north to south increasing gradient [6]. Malaria transmission is highly seasonal and peaks during the rainy season, but it has been shown that transmission can continue late into the dry season [6–9]. Assessing *P. falciparum* genetic diversity may be useful to elucidate the mechanisms of transmission persistence and rebound. Such studies would provide insight into human reservoirs of the parasites, including symptomatic cases and those of asymptomatic carriage [3, 8, 10] and human migration patterns associated with parasite flux [11–15]. Additionally, a clear understanding of *P. falciparum* genetic diversity would shed light on the characteristics of malaria burden and the expected difficulties hindering malaria control. Indeed, it has been shown that *P. falciparum* genetic diversity is indicative of the ability of malaria parasites to adapt to their hosts by selection of advantageous traits, such as drug resistance and antigenic variability [16].

*Plasmodium falciparum* genetic diversity can be assessed by analysing genetic polymorphism of the merozoite surface proteins (*msp*-*1* and *msp*-*2*), as previously published in Mali [8, 10, 17, 18]. These markers were useful to investigate genetic diversity, multiplicity of infections and parasite carriage. However, the *msp*-*1* and *msp*-*2* genes are under selective pressure and neutral markers such as microsatellites or single nucleotide polymorphism (SNPs) are better suited for population genetics assessment [19]. Highly polymorphic microsatellite markers have been widely used to study *P. falciparum* population genetics via multiple loci variable number of tandem repeats analysis (MLVA) [11–15, 20–23]. These studies provided insights into various *P. falciparum* population genetic features, including parasite migration and linkage disequilibrium. To date, microsatellite markers have never been assessed to study *P. falciparum* population genetics in Mali.

In this study, MLVA was performed on *P. falciparum* DNA extracted from blood samples collected from four Malian study sites displaying various malaria transmission patterns. The four study sites were located along a 900-km long north to south axis and included the city of Bamako.

## Methods

### Study sites

**Fig. 1:**
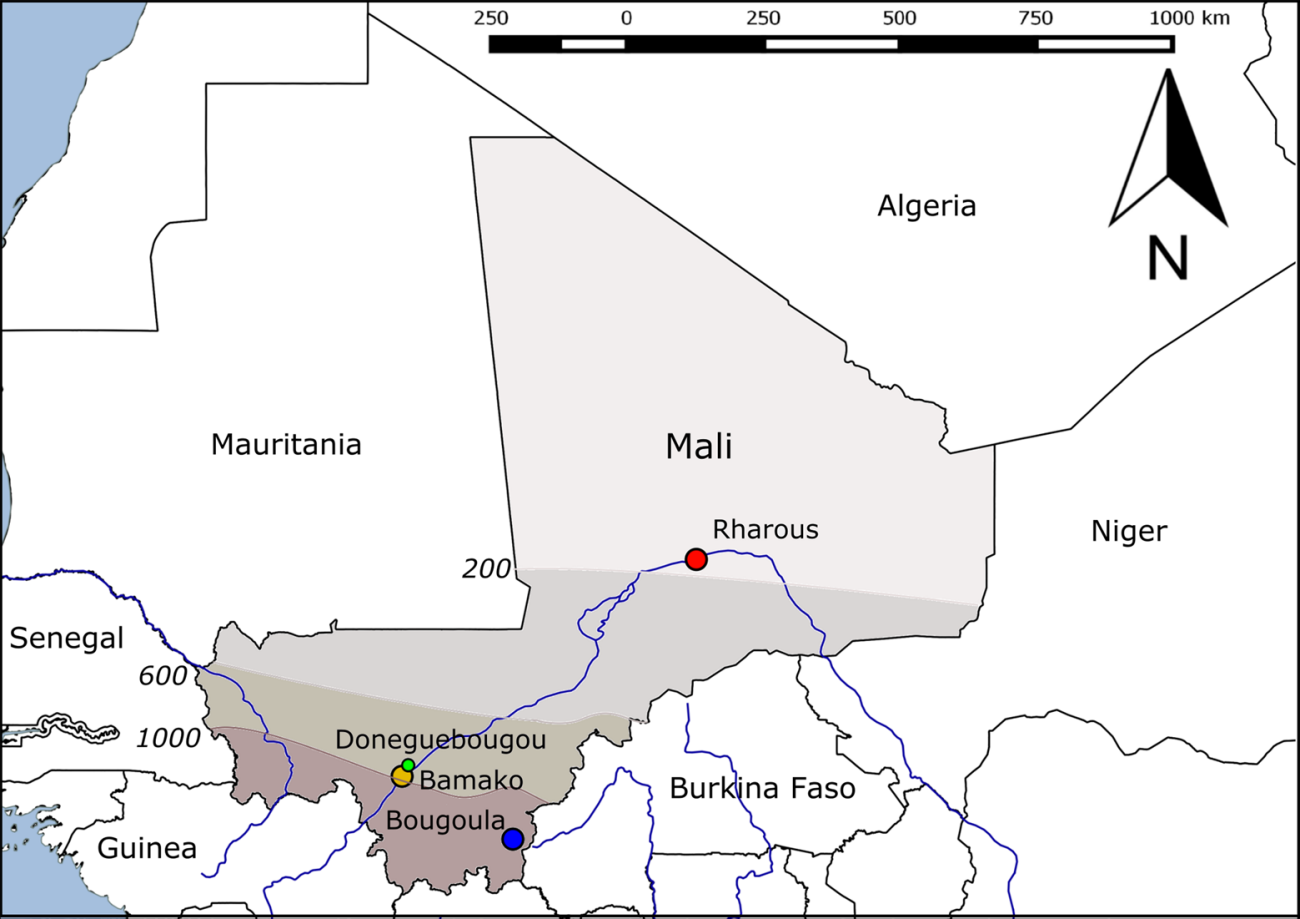
Maps of Mali showing four study sites and four malaria epidemiological patterns [6, 24]. Annual isohyets (mm) separate each climatic zone. The climatic zones from north to south are as follows: Saharian zone (malaria transmission is sporadic to epidemic), Sahelian and Sudano-Sahelian zones (hyper-endemic for malaria, short transmission season of 3–4 months), Sudano-Guinean zone (hyper-endemic for malaria, long transmission season of 4–6 months). In the urban area of Bamako, conditions are not favourable for malaria transmission (hypo-endemic malaria)

Blood samples were collected in Rharous (Timbuktu District), Bamako (Bamako District), Doneguebougou (Kati District), and Bougoula Hameau (Sikasso District) (Fig. 1). Each of the study sites represents a different pattern of malaria transmission as defined by previous Malian epidemiological reports [6, 24, 25]. According to these reports, malaria is hypo-endemic in the urban zone of Bamako, whereas the disease is sporadic with occasional epidemics in Rharous, which is located in the Sahara Desert. Malaria is hyper-endemic in Doneguebougou [a site located in the Sudano-Sahelian zone, where malaria is characterized by a short transmission season (3–4 months)] and Bougoula [which is located in the Sudano-Guinean zone, where the transmission season is longer (4–6 months)].

### Study design

A total of 648 blood samples were collected by finger prick (~50 µL) and adsorbed onto filter paper (Whatman™ 3MM CHR). Dried blood spots on filter papers were transported and stored at ambient temperature at the Malaria Research and Training Centre (MRTC) Central Laboratory in Bamako, Mali. Along with blood sampling, epidemiological and clinical data were reported on a standardized form. Sample collection was performed at the end of the rainy season (October) and at the beginning of the dry season (January to March), which correspond to the highest malaria transmission periods [7] (Table 1). Samples were collected via active case detection, with the exception of Rharous, where passive case detection was employed due to political instability in this area (Table 1). Active case detection included both symptomatic and asymptomatic individuals for malaria, whereas passive case detection included only individuals presenting with a fever or reported fever to the community health centre staffed by the MRTC. Demographic and clinical features of the cohort are detailed in Table 1.

**Table 1:** Demographic and clinical features of each cohort, four sites in Mali

	Rharous, n = 149	Bamako, n = 249	Doneguebougou, n = 100	Bougoula, n = 150	*P* value
Malaria endemicity	Sporadic	Hypo-endemic	Hyper-endemic	Hyper-endemic	
Period of collection	December 2012–March 2013	October 2013	October 2013	October 2013	
Season	Dry (beginning)	Rainy	Rainy	Rainy	
Detection method^a^	PCD	ACD	ACD	ACD	
Female (%)	54/149 (64)	132/249 (53)	51/100 (51)	96/150 (64)	0.11
Mean age ± SD	23.82 ± 17.34	20.43 ± 18.10	16.68 ± 15.31	31.30 ± 25.26	<0.0001
Children (0–5 years old) (%)	25/145 (17)	49/249 (20)	27/100 (27)	39/148 (26)	
Children (6–18 years old) (%)	42/145 (29)	97/249 (39)	41/100 (41)	24/148 (16)	
Adults (>18 years old) (%)	78/145 (54)	103/249 (41)	32/100 (32)	85/148 (57)	<0.001
Fever^b^ (%)	44/137 (32)	38/249 (15)	8/99 (8)	18/149 (12)	<0.0001

^a^ *ACD* active case detection; *PCD* passive case detection

^b^ *Fever* axillary temperature ≥37.5 °C

### Ethical approval

Ethical clearance was obtained from the Ethics Committee of the Faculty of Medicine of Bamako (No 2012/81/CE/FMPOS) and blood samples were obtained after individuals provided informed consent.

### *Plasmodium falciparum* isolation and quantification

#### DNA extraction

Filter papers with dried blood spots were cut into two pieces of approximately 1 cm^2^ using scissors and were incubated for 48 h at room temperature in 800 µL EasyMAG lysis buffer (bioMérieux, Marcy l’Étoile, France). Nucleic acid extraction was then performed using a NucliSENS EasyMAG system (bioMérieux, Marcy l’Étoile, France) with an elution volume of 100 µL [26]. Screening for the presence of *P. falciparum* was performed using specific primers and probe targeting the 18S *rRNA* gene via qPCR with a LightCycler 480 PCR system (Roche Diagnostics, Meylan, France) as previously described [27]. Each experimental run included both a negative (no template) and a positive (plasmid of *P. falciparum*18S *rRNA*) control. Standard curves were generated from serial ten-fold dilutions of the plasmid for species-specific quantification of parasite density (number of parasites/μL of blood). It was assumed that each genome of *P. falciparum* has five copies of the *18S rRNA* gene (as observed in the 3D7 genome) [28].

#### MLVA genotyping

Genotyping relied on the analysis of eight polymorphic microsatellite markers (Poly α, TA109, TA1, TA81, TA42, ARA2, PfPK2, Pfg377) distributed among five chromosomes of *P. falciparum*, as previously published [20, 29]. Microsatellites were amplified via a two-step, semi-nested PCR using fluorescent end-labelled primers as previously described [29, 30]. Primer sequences, chromosome location, as well as reaction and cycling conditions are detailed in Additional file 1. To determine repeat-length sizes, PCR products were analysed using an ABI 3130xl capillary sequencer (Applied Biosystems, Foster City, CA, USA). Microsatellite allele length was determined using internal size standards (GeneScan 500 LIZ Size Standard, Applied Biosystems) with the GeneMapper v4.0 software (Applied Biosystems). To differentiate allele peaks from stutter peak artefacts, multiple alleles per locus were scored if minor peaks were >33 % of the height of the major peak, corresponding to the predominant allele [20, 29]. Peaks with a fluorescence intensity <100 units were discarded.

### Population genetics analysis

Only complete genotypes (presenting all eight loci) were included in the population genetics analysis (complete dataset).

### Multiplicity of infection

Multiplicity of infection (MOI) is defined as the number of genetically distinct clones of *P. falciparum* (i.e., number of distinct parasite genomes) present in an individual. As *P. falciparum* is haploid in the human stage, multiple peaks or alleles correspond to an infection with multiple genotypes or strains (a polygenomic infection). In a given sample, MOI was scored as the maximum number of alleles observed when taking into account all analysed loci (n = 8). An average MOI was then calculated.

### Measure of genetic diversity

Because of the frequent occurrence of polygenomic infections, only major peaks were considered to assemble genotypes for genetic diversity analysis, as previously described [11, 12, 15, 20, 21]. The robustness of the genotyping assay was tested using two datasets: (1) built with major peaks only (first dataset); and, (2) built with minor peaks only (second dataset). The dataset including the major peaks only was used, as no significant difference in results has been observed. Allelic diversity is usually measured using expected heterozygosity (He). As *P. falciparum* is haploid in the human host, the terms ‘genetic diversity’ and ‘Nei’s unbiased genetic diversity index’ were employed instead of ‘expected heterozygosity (He)’. Nei’s unbiased genetic diversity index was calculated using ARLEQUIN v.3.5 [31] with the following formula:

$$He = \left[\left( {\frac{n}{n - 1}} \right) \times \left( {1 - \mathop \sum \nolimits p^{2} } \right)\right]$$

in which *n* = number of samples analysed and *p* = frequency of each allele at a given locus [32]. For a haploid organism, Nei’s genetic diversity index can be considered as a measure of the probability to randomly draw a pair of different alleles from an allelic pool. Potential values range from 0 (no diversity, 100 % similarity between alleles) to 1 (maximal diversity, 100 % of the alleles are different). Allelic richness, which is an index derived from the normalization of the number of alleles with respect to sampling size, was calculated for each site using FSTAT v.2.9.3.2 [33].

### Population genetic structure

The complete dataset, including genotypes from the four study sites, was analysed to calculate Wright’s F-statistics (F_ST_), a measure of genetic differentiation between populations, using ARLEQUIN v 3.5 [31].

### Linkage disequilibrium

The overall multi-locus linkage disequilibrium (LD) was assessed using the LIAN v.3.7 web interface [34]. This genetic phenomenon follows recurrent recombination between alleles at distinct loci instead of a random association of alleles. LIAN compares the variance in the number of alleles shared between all pairs of genotypes with the variance expected under a random association hypothesis and calculates the Standard Index of Association (*I*_*S*_^*A*^), a quantitative measure of LD. Values range from 0 (no loci in LD) to 1 (all loci in LD).

#### Genotype analysis

A minimum spanning tree was constructed based on the complete dataset to investigate genetic connections between genotypes. The chosen plug-in and the cluster analysis tool were implemented in BioNumerics v7.1 Software (Applied Maths, Ghent, Belgium).

### Statistical analysis

Parasite densities of the four sites were compared using a negative binomial regression model (Genmod procedure). The influence of categorical variables including gender, age category (up to 5 years, 6–18 years, >18 years) and fever on the occurrence of polygenomic infections was assessed via Chi squared test (Chi^2^). *Plasmodium falciparum* MOI association with sex, age group, study site, and parasite density was tested via univariate unconditional logistic regression analysis (Logistic procedure). Multivariate unconditional logistic regression analysis with stepwise selection was performed to select the most parsimonious, best-fitting model of *P. falciparum* MOI. The analyses were performed using two-sided tests with a *P* < 0.05 significance level using SAS v9.2 statistical software (SAS Institute Inc, Cary, NC, USA).

## Results

### PCR results

**Fig. 2:**
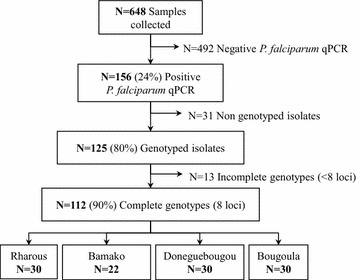
Study flow chart of samples collected from the four sites in Mali. Positive qPCR samples were genotyped until 30 complete genotypes (successfully genotyped for all tested loci) were obtained for each study site. In the site of Bamako, only 22 complete genotypes were obtained due to relatively low case numbers

The *P. falciparum* qPCR was positive in 156 of 648 tested samples (see study flow chart on Fig. 2). Positivity rate of *P. falciparum* qPCR differed significantly between the different locations, ranging from 9 % in Bamako to 44 % in Doneguebougou (*P* < 0.0001, Table 2). Positive qPCR samples were genotyped until 30 complete genotypes (i.e., successfully genotyped for all eight tested loci) were obtained for each study site. However, in Bamako, only 22 complete genotypes were obtained overall due to low malaria incidence. Of 125 genotyped samples, 112 genotypes were complete (Fig. 2). The results of the *P. falciparum* qPCR and estimated parasite density for each study site are detailed in Table 2. The microsatellite dataset is included in Additional file 2.

**Table 2:** *Plasmodium falciparum* qPCR results, estimated parasite density and polygenomic infections, four sites in Mali

	Rharous	Bamako	Doneguebougou	Bougoula	*P* value
Total					
Positive qPCR for *P. falciparum* (%)	44/149 (30)	23/249 (9)	44/100 (44)	45/149 (30)	<*0.0001*
Polygenomic infections^d^ (%)	11/30 (37)	9/22 (41)	21/30 (70)	25/30 (83)	<*0.0001*
Mean MOI^c^	1.4^e^	1.5^f^	1.9	2.06	<*0.001*
Mean estimated parasitaemia ± SD^b^ (parasites/µL blood)	4397 ± 8127	5348 ± 5209	2942 ± 5863	8755 ± 24,395	0.061
Individuals with fever^a^					
Positive qPCR for *P. falciparum* (%)	22/44 (50)	13/38 (34)	6/8 (75)	12/18 (66)	0.051
Polygenomic infections^d^ (%)	9/16 (56)	5/13 (38)	3/5 (60)	6/8 (75)	<*0.0001*
Mean MOI^c^	1.62	1.46	1.6	1.75	0.72
Mean estimated parasitaemia ± SD^b^ (parasites/µL blood)	7335 ± 10639	6916 ± 5934	9063 ± 12,318	5731 ± 9192	0.86
Apyretic individuals^a^					
Positive qPCR for *P. falciparum* (%)	20/93 (21)	10/211 (5)	38/91 (42)	32/131 (24)	<*0.0001*
Polygenomic infections^d^ (%)	2/14 (14)	4/9 (44)	18/25 (72)	19/22 (86)	<*0.0001*
Mean MOI^c^	1.14	1.55	1.96	2.18	<*0.0001*
Mean estimated parasitaemia ± SD^b^ (parasites/µL blood)	1368 ± 2066	3311 ± 3348	1976 ± 3523	10148 ± 28,407	<*0.01*

Differences in the denominator are due to missing data (n = 14 missing axillary temperature data)

Statistically significant results are indicated in italic

^a^Fever: axillary temperature ≥37.5 °C and apyretic: axillary temperature <37.5 °C

^b^Estimation of parasite densities: [(copy number of the gene in 1 µL of DNA) × (100/125)]. Assuming that each genome of *P. falciparum* has five copies of the 18S *rRNA* gene [28], that blood volume spotted onto filter papers was 25 µL (half filter paper) and that extracted DNA was eluted into 100 µL

^c^MOI was calculated based on complete genotypes

MOI calculated in each site = ∑(maximum number alleles for a given sample) ÷ ∑(genotyped samples)

^d^Polygenomic infections are defined as individuals infected by ≥2 distinct parasite genotypes

^e^Statistically significant compared with Doneguebougou and Bougoula

^f^Statistically significant compared with Bougoula

### Multiplicity of infection (MOI)

The proportion of polygenomic infections and mean MOI values per study site are shown in Table 2. Mean proportion of polygenomic infections was 58.9 % of the total cohort. The mean MOI significantly differed according to the study site (*P* < 0.001). Mean MOI values ranged from 1.4 (Rharous) to 2.06 (Bougoula) and were intermediate in Bamako (1.5). Similarly, polygenomic infection prevalence significantly differed based on the study sites (*P* < 0.0001). The lowest polygenomic infection rate was observed in Rharous (37 %), while the highest rate was noted in Bougoula (83 %). Univariate analysis revealed no significant effect of gender, age, fever, and parasite density on the occurrence of *P. falciparum* polygenomic infections (Table 3). In contrast, univariate analysis confirmed that the risk of polygenomic infection was significantly associated with study site (*P* < 0.001). Accordingly, the risk was significantly lower in Rharous (*P* < 0.01) and Bamako (*P* < 0.05) when compared with Bougoula.

**Table 3:** Univariate logistic analysis of *Plasmodium falciparum* polygenomic infections

	Proportion of monogenomic infections (%)	Proportion of polygenomic infections (%)	OR	95 % CI	*P* value
Female	24/57 (42)	33/57 (58)	0.917	[0.432–1.947]	0.82
Age group					0.19
Children (0–5 years old)	6/23 (26)	17/23 (74)	3.051	[0.921–10.114]	0.083
Children (6–18 years old)	26/62 (42)	36/62 (58)	1.491	[0.601–3.697]	0.69
Adults (>18 years old)	14/27 (52)	13/27 (48)	1	–	–
Fever (axillary temp ≥37.5 °C)	19/42 (45)	23/42 (55)	0.760	[0.350–1.650]	0.49
Ln (parasite density)	–	–	1.063	[0.823–1.373]	0.64
Site					<*0.001*
Rharous	19/30 (63)	11/30 (37)	0.116	[0.034–0.390]	<*0.01*
Bamako	13/22 (59)	9/22 (41)	0.138	[0.038–0.499]	<*0.05*
Doneguebougou	9/30 (30)	21/30 (70)	0.467	[0.135–1.609]	0.19
Bougoula	5/30 (17)	25/30 (83)	1	–	Reference

Statistically significant results are indicated in italics

### Genetic diversity

Genetic diversity values (He) for each study site are shown in Table 4. Genetic diversity values were also high between the different study sites (He = 0.76–0.79; mean He ± SD = 0.77 ± 0.16).

**Table 4:** Genetic diversity of *Plasmodium falciparum* at the four study sites in Mali

Locus	Rharous, n = 30		Bougoula, n = 30		Doneguebougou, n = 30		Bamako, n = 22	
	A	He	A	He	A	He	A	He
PolyA	13.51	0.92	13.45	0.91	14.37	0.91	9.00	0.84
TA109	8.72	0.85	9.66	0.86	7.86	0.85	12.00	0.93
Pfpk2	10.58	0.88	9.72	0.87	6.86	0.80	7.00	0.85
ARA2	6.85	0.71	5.92	0.75	8.78	0.85	7.00	0.82
Pfg377	3.00	0.54	4.92	0.68	4.86	0.63	4.00	0.60
TA42	3.86	0.43	3.86	0.35	4.79	0.56	4.00	0.46
TA81	8.72	0.83	7.72	0.78	6.79	0.79	7.00	0.73
TA1	11.52	0.88	9.79	0.89	8.92	0.90	9.00	0.86
All loci								
Mean	8.34	0.76	8.13	0.76	7.90	0.79	7.37	0.76
SD	3.64	0.17	3.14	0.18	3.04	0.12	2.66	0.15

*A* Allelic richness based on minimum sample size of 22 individuals (Bamako)

*He* Nei’s unbiased genetic diversity index

### Population structure between geographic sites

Table 5 shows the pairwise F_ST_ between the four study sites, which reveals an almost complete panmixia with very low F_ST_ values (*F*_ST_ = 0.0011–0.018). Pairwise *F*_ST_ was statistically significant only between Bamako and Rharous (*P* = 0.03).

**Table 5:** Matrix of between study site pairwise* F*_ST_

	Rharous	Bougoula	Doneguebougou
Bougoula	0.0011	–	–
Doneguebougou	0.0065	0.0072	–
Bamako	0.018*	0.011	0.0026

* P value = 0.03

### Linkage disequilibrium (LD)

Global multi-locus LD analysis of genotypes showed an absence of significant LD among alleles of *P. falciparum* genotypes in the different study sites. The *I*_*S*_^*A*^ values and associated *P* values are shown in Table 6.

**Table 6:** Multilocus linkage disequilibrium analysis of *Plasmodium falciparum* genotypes

	*I*_*S*_^*A*^	*P* value
Rharous	−0.0015	0.54
Bougoula	0.0065	0.25
Doneguebougou	0.0005	0.48
Bamako	−0.0071	0.72

### Genotype analysis

**Fig. 3:**
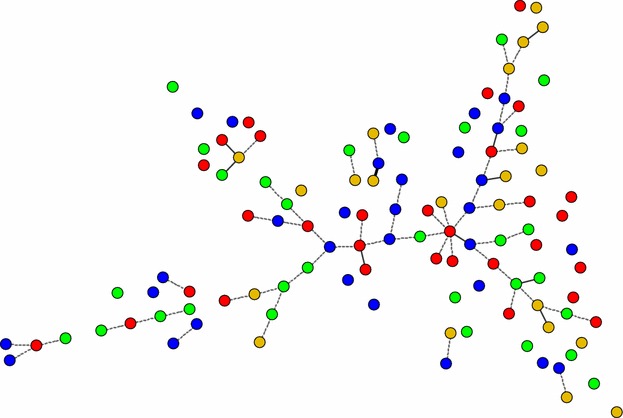
Minimum spanning tree showing the genetic relationship between 112 *Plasmodium falciparum* genotypes. Each genotype was unique. The *thick solid lines* indicate that the connected genotypes differ by only one locus of microsatellite marker, i.e., single-locus variant. Similarly, the *thin solid lines* represent double-loci variants and the *dotted lines* indicate triple-loci variants. Therefore, unlinked genotypes differ by more than three loci variants of the tested microsatellite markers. Isolates are coloured-coded according to geographic location in the dendrogram as follow: Rharous (*red*), Bougoula (*blue*), Doneguebougou (*green*), Bamako (*gold*). The distance between genotypes in the diagram does not reflect any relationship with genetic distance between genotypes

Of the 112 complete genotypes, 100 % were unique genotypes as they were found only in a single sample. Most genotypes differed by two, three or more than three loci variants of the microsatellite markers. No major clustering of genotypes was detected between and within study sites as shown in Fig. 3.

## Discussion

This work represents the most extensive investigation of genetic diversity of *P. falciparum* populations in Mali to date and the first study to employ MLVA genotyping of *P. falciparum* in this country. *Plasmodium falciparum* isolates from Mali displayed high genetic diversity (mean He = 0.77) and low genetic differentiation (*F*_ST_ < 0.02). The lack of LD further revealed an absence of local clonal expansion, which was corroborated by the genotype relationship results. These findings highlight the marked gene flux between parasite populations, including within sites of lower transmission rates (Rharous and Bamako) and between sites located 900 km from each other (Rharous and Bougoula). In contrast with the lack of variability between genetic diversity levels observed in the country, mean MOI and proportions of polygenomic infection showed a north to south increasing gradient paralleling local malaria transmission levels [6].

Comparable levels of genetic diversity (He = 0.72–0.8) have already been observed in multi-locus microsatellite studies of *P. falciparum* in African regions highly endemic for malaria [11, 12, 20, 21]. This African pattern differs dramatically to those observed in hypo-endemic Latin American countries where genetic diversity is low (He = 0.30–0.40) [20, 23, 35] and Southeast Asia, where genetic diversity is intermediate (He = 0.51–0.65), which is probably due to more advanced malaria control progress in these areas [13, 20]. Indeed, the lower genetic diversity levels in Latin America and Southeast Asia, compared with Africa, might stem from a reduction in parasite population size following a decline in malaria transmission rates [20, 36].

Genetic diversity metrics have been useful to assess malaria control interventions and to highlight the disparities in malaria control programme results in endemic African countries [5, 11, 12, 15, 20, 21, 37]. The present study confirms the high genetic diversity previously reported in analyses of genetic polymorphism of the merozoite surface proteins (*msp*-*1* and *msp*-*2*) [8, 10, 17, 18] and further indicates an absence of local clonal expansion in Mali. In Sudan, it has also been reported that *P. falciparum* genetic diversity remained high despite bolstered malaria control efforts [37]. In contrast, declining rates of genetic diversity and oligo-clonal expansion of parasite populations have been reported during a longitudinal study of *P. falciparum* microsatellite markers in Djibouti between 1998 and 2009 [15]. This setting was compatible with pre-elimination objectives. Comparable results of *P. falciparum* clonality have also been observed in Senegal (Thies city) during a longitudinal study using a set of 24 SNPs molecular barcode [5]. Clonal transmission of malaria has been detected after enhanced interventions, thereby revealing a dramatic change in the population structure. Such differences in *P. falciparum* population genetics in African countries suggest that in Mali, malaria control interventions still exert a limited impact on *P. falciparum* populations and malaria transmission.

Findings highlight pronounced gene flux between parasite populations. As illustrated in Rharous (northern Mali), the association between a high proportion of monogenetic infections (63 %) and high genetic diversity (He = 0.76) suggest that genetically diverse parasite strains have been imported from highly endemic regions via extensive human migration events. A similar trend was observed in the capital city of Bamako, thereby suggesting that many malaria cases are being imported from rural areas. These hypotheses are also supported by the lack of genetic differentiation between the different study sites. In Nigeria [12], a lack of genetic differentiation between *P. falciparum* populations located approximately 200 km apart was depicted as the marker of substantial parasite flux between the two locations. In the present study, a similar pattern was observed between study sites located up to 900 km apart (Rharous and Bougoula). A possible explanation could be the serious political crisis that occurred in 2012 and 2013 in Mali, which led to significant deterioration of security in the northern regions as well as displacement of large populations to neighbouring countries and the southern areas of Mali. Importantly, these findings imply that the effectiveness of malaria control efforts may be impaired if all malaria foci are not tackled simultaneously. High parasite flux from one site to another may also favour the diffusion of anti-malarial drug-resistant strains.

The analysis of the relationship between genotypes further revealed that each *P. falciparum* genotype was unique, with an absence of population clustering, even in the remote low-transmission site of Rharous. Human migration events might play a role in this phenomenon by boosting genetic diversity amongst *P. falciparum* populations. The absence of local clonal expansion was further corroborated by the lack of LD, which revealed a panmixia parasite population. Nevertheless, other markers, such as SNPs, might be more prone to show clonal expansion of parasites [19]. For instance, in Senegal, which is adjacent to Mali, a local clonal expansion of *P. falciparum* populations has been observed in Thies city using SNP molecular barcode [3, 5]. Therefore, it would be interesting to complete this study, especially in the hypo-endemic site of Rharous, to determine to which extent the absence of local clonal expansion can be confirmed using SNP markers.

The lowest levels of MOI and proportion of polygenomic infections were found in the areas exhibiting low malaria transmission, namely the urban site of Bamako and the Sahelo-Saharan site of Rharous. In contrast, the highest levels were found in the Sudano-Guinean site of Bougoula, where malaria transmission is almost perennial. A similar trend has been observed in two Malawian districts characterized by different transmission settings [38]. Although the association between prevalence of polygenomic infections and age group has been shown [10, 38], a longitudinal study in Mali [10] found that the increase in prevalence of polygenomic infections affected all age groups during the peak malaria transmission season, which is concordant with the results of the present study.

A limitation of this study may be that the human cohorts of each study site were not totally comparable. Indeed, a passive sampling procedure was used in Rharous due to security issues that precluded active case detection (Table 1). This explains why the proportion of febrile malaria cases in Rharous was significantly higher than in the other sites (*P* < 0.0001). Nevertheless, the significantly lower proportion of polygenomic infections observed in Rharous is probably not due to differences in case detection, as the univariate analysis revealed no association between proportion of polygenomic infections and fever, patient sex, age group, or estimated parasitaemia. Variations in proportion of polygenomic infections correlated with the study site only (*P* < 0.001). Additionally, disparities between malaria cohorts, such as fever prevalence, seem not to have impacted genetic diversity level, as it was similar in the different study sites. Likewise, this observation was further supported by a comparable high level of allelic richness.

## Conclusions

Overall, these results reveal that *P. falciparum* genetic diversity level is particularly high in Mali. Such diversity may constitute an obstacle to control malaria as genetic polymorphism may facilitate parasite populations to adapt to their host and counteract certain interventions, such as anti-malarial vaccination. The pronounced parasite gene flux mediated by human migration events likely represents another hindrance for malaria control. Indeed, high parasite flux from one site to another could favour the diffusion of parasite strains, including those resistant to anti-malarial drugs. Therefore, these findings argue that malaria control measures should be intensified in Mali and that all malaria foci must be tackled simultaneously to reduce *P. falciparum* genetic diversity. As the measures currently in place (ACT and LLINs) seem to have a limited impact on *P. falciparum* populations, malaria control programmes aiming at reducing both malaria burden and parasite populations are greatly warranted.

## Additional files

10.1186/s12936-016-1397-0 Primer sequences of eight *Plasmodium falciparum* microsatellite loci, chromosome location and PCR reaction conditions. As previously described [29], primers listed third are internal to the other two and are end-labelled (*). PCR master mixes were comprised of 12 µL of Light Cycler 480 Probes Master (Roche Diagnostics, Meylan, France), 0.5 µL of each primer at 10 µM (Eurogentec, Seraing, Belgium), (Applied Biosystems, Foster, USA) and 1 µL of DNA. Thermocycling was performed in a 96-well T3 thermocycler (Biometra, Goettingen, Germany). For the first PCR reaction, the PCR programme was as follows: denaturation at 94 °C for 2 min, 1 cycle; (denaturation at 94 °C for 30 s, annealing at 42 °C for 30 s, annealing at 40 °C for 30 s, final elongation at 65 °C for 40 s), 25 cycles; extension time of 2 min at 65 °C, 1 cycle. For the second PCR reaction, the PCR programme was as follows: denaturation at 94 °C for 2 min, 1 cycle; (denaturation at 94 °C for 20 s, annealing at 45 °C for 30 s, final elongation at 65 °C for 30 s), 25 cycles; extension time of 2 min at 65 °C, 1 cycle.
10.1186/s12936-016-1397-0 Microsatellite data of the genotyped samples. The allele length (in base pairs) for each *Plasmodium falciparum* tested loci (Poly α, TA109, TA1, TA81, TA42, ARA2, PfPK2, Pfg377) is provided. The empty cells of the Table correspond to negative loci.

## Abbreviations

RDT: rapid diagnostic test
MLVA: multiple loci variable number of tandem repeats analysis
SNP: single nucleotide polymorphism
MRTC: Malaria Research and Training Centre
ACT: artemisinin-based combination therapy
LLIN: long-lasting insecticide-treated bed net
MOI: multiplicity of infection
LD: linkage disequilibrium
