# Supplementary material for: Genetic diversity of Plasmodium falciparum in human malaria cases in Mali
Source: Malar J. 2016 Jul 11;15:353. doi: 10.1186/s12936-016-1397-0 (PMC4940954; doi:10.1186/s12936-016-1397-0)
Supplement: Supplementary file 1 — 10.1186/s12936-016-1397-0 Primer sequences of eight Plasmodium falciparum microsatellite loci, chromosome location and PCR reaction conditions. As previously described [29], primers listed third are internal to the other two and are end-labelled (*). PCR master mixes were comprised of 12 µL of Light Cycler 480 Probes Master (Roche Diagnostics, Meylan, France), 0.5 µL of each primer at 10 µM (Eurogentec, Seraing, Belgium), (Applied Biosystems, Foster, USA) and 1 µL of DNA. Thermocycling was performed in a 96-well T3 thermocycler (Biometra, Goettingen, Germany). For the first PCR reaction, the PCR programme was as follows: denaturation at 94 °C for 2 min, 1 cycle; (denaturation at 94 °C for 30 s, annealing at 42 °C for 30 s, annealing at 40 °C for 30 s, final elongation at 65 °C for 40 s), 25 cycles; extension time of 2 min at 65 °C, 1 cycle. For the second PCR reaction, the PCR programme was as follows: denaturation at 94 °C for 2 min, 1 cycle; (denaturation at 94 °C for 20 s, annealing at 45 °C for 30 s, final elongation at 65 °C for 30 s), 25 cycles; extension time of 2 min at 65 °C, 1 cycle. [file 12936_2016_1397_MOESM1_ESM.pptx]

## Slide 1
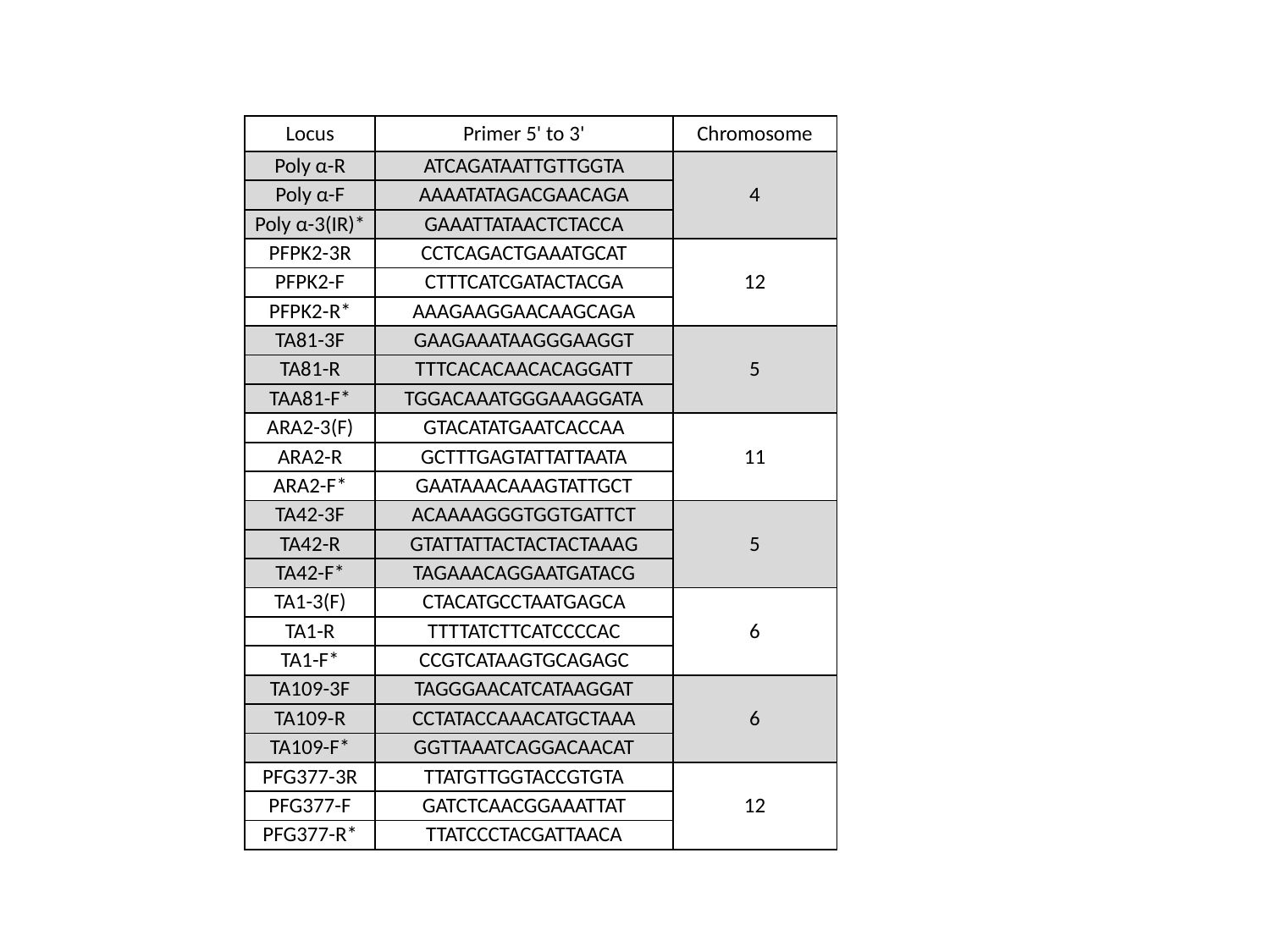

| Locus | Primer 5' to 3' | Chromosome |
| --- | --- | --- |
| Poly α-R | ATCAGATAATTGTTGGTA | 4 |
| Poly α-F | AAAATATAGACGAACAGA | |
| Poly α-3(IR)\* | GAAATTATAACTCTACCA | |
| PFPK2-3R | CCTCAGACTGAAATGCAT | 12 |
| PFPK2-F | CTTTCATCGATACTACGA | |
| PFPK2-R\* | AAAGAAGGAACAAGCAGA | |
| TA81-3F | GAAGAAATAAGGGAAGGT | 5 |
| TA81-R | TTTCACACAACACAGGATT | |
| TAA81-F\* | TGGACAAATGGGAAAGGATA | |
| ARA2-3(F) | GTACATATGAATCACCAA | 11 |
| ARA2-R | GCTTTGAGTATTATTAATA | |
| ARA2-F\* | GAATAAACAAAGTATTGCT | |
| TA42-3F | ACAAAAGGGTGGTGATTCT | 5 |
| TA42-R | GTATTATTACTACTACTAAAG | |
| TA42-F\* | TAGAAACAGGAATGATACG | |
| TA1-3(F) | CTACATGCCTAATGAGCA | 6 |
| TA1-R | TTTTATCTTCATCCCCAC | |
| TA1-F\* | CCGTCATAAGTGCAGAGC | |
| TA109-3F | TAGGGAACATCATAAGGAT | 6 |
| TA109-R | CCTATACCAAACATGCTAAA | |
| TA109-F\* | GGTTAAATCAGGACAACAT | |
| PFG377-3R | TTATGTTGGTACCGTGTA | 12 |
| PFG377-F | GATCTCAACGGAAATTAT | |
| PFG377-R\* | TTATCCCTACGATTAACA | |
